# An Analysis of the Mixed IEEE 802.11ax Wireless Networks in the 5 GHz Band

**DOI:** 10.3390/s23104964

**Published:** 2023-05-22

**Authors:** Marek Natkaniec, Natalia Bieryt

**Affiliations:** Institute of Telecommunications, AGH University of Krakow, al. Mickiewicza 30, 30-059 Krakow, Poland; bieryt.natalia@gmail.com

**Keywords:** IEEE 802.11ax, legacy devices, mixed networks, BSS coloring, performance evaluation

## Abstract

This paper presents an analysis of the IEEE 802.11ax networks’ coexistence with legacy stations, namely IEEE 802.11ac, IEEE 802.11n, and IEEE 802.11a. The IEEE 802.11ax standard introduces several new features that can enhance network performance and capacity. The legacy devices that do not support these features will continue to coexist with newer devices, creating a mixed network environment. This usually leads to a deterioration in the overall performance of such networks; therefore, in the paper, we want to show how we can reduce the negative impact of legacy devices. In this study, we investigate the performance of mixed networks by applying various parameters to both the MAC and PHY layers. We focus on evaluating the impact of the BSS coloring mechanism introduced to the IEEE 802.11ax standard on network performance. We also examine the impact of A-MPDU and A-MSDU aggregations on network efficiency. Through simulations, we analyze the typical performance metrics such as throughput, mean packet delay, and packet loss of mixed networks with different topologies and configurations. Our findings indicate that implementing the BSS coloring mechanism in dense networks can increase throughput by up to 43%. We also show that the presence of legacy devices in the network disrupts the functioning of this mechanism. To address this, we recommend using an aggregation technique, which can improve throughput by up to 79%. The presented research revealed that it is possible to optimize the performance of mixed IEEE 802.11ax networks.

## 1. Introduction

There has been an exponential rise in the number of wireless devices in recent years. According to the statistical data published by Cisco, by 2023, the number of wireless access points will increase by almost four times compared to 2018. It is also estimated that the number of next-generation access points will increase 13-fold by 2023, which will account for 11% of the total [1]. Undoubtedly, new solutions in the field of wireless transmission techniques will have a crucial impact on the delivery of efficient network services. The huge increase in the number of devices and sensors has led to the emergence of so-called dense environments, which include residential blocks, shopping centers, stadiums, and concert halls, among others. Scientists aiming to improve the IEEE 802.11 standard have had to deal with these problems and provide users with the appropriate throughput and low delays [2]. The main focus was on improving the efficiency of the entire network, not just individual stations, by increasing spectral efficiency and using mechanisms to achieve better traffic management. As a result, in 2020, the latest extension, IEEE 802.11ax, was approved by the Task Group 802.11ax, which is also known as Wi-Fi 6th generation [3]. This is the first such extension that focuses on dense networks [4]. Dense networks can enable seamless communication and data transmission among IoT devices and sensors, facilitating the deployment of various smart applications.

IEEE 802.11ax has introduced many new functionalities that can significantly improve transmission quality metrics. However, upgrading all client devices to the latest Wi-Fi standard is a time-consuming process, which lasts for many years. Thanks to its provision of backward compatibility, the IEEE 802.11ax standard can cooperate with devices based on previous extensions (known as legacy stations). Stations that use older extensions of the standard, although they cannot use the mechanisms introduced by IEEE 802.11ax, can also benefit from connecting to a next-generation access point that uses new radio techniques. Additionally, networks based on the latest extension, by offering wider radio channels, can provide legacy stations with more opportunities for transmission by allowing for more frequent and parallel access to the radio channels, which results from their aim to effectively utilize the available bandwidth.

Recent scientific studies have demonstrated that the efficiency of IEEE 802.11 networks using the latest extensions can be significantly impaired by the presence of legacy devices. The aim of this work is to analyze and optimize the performance of IEEE 802.11ax networks during their coexistence with legacy stations by applying various parameters to both the MAC and PHY layers. The research focuses particularly on evaluating the newly introduced network BSS coloring mechanism, which strives to enhance performance in scenarios where multiple networks coexist on the same channel by assigning a distinct identifier, known as a color, to each of them. To determine the optimal parameters that affect quality metrics, such as throughput, delay, and packet loss, various network topologies and configurations were simulated. The impact of a percentage change in the number of legacy nodes on the throughput achieved by the network was also evaluated. An analysis of the obtained results allowed for the parameters and conditions of the network that have the greatest impact on its performance to be determined. Our research demonstrates that achieving widespread and efficient wireless access may not be feasible if novel and legacy Wi-Fi devices are not properly configured. The main contributions of this paper are as follows:

- We analyzed the impact of the number of coexisting BSS networks on the throughput gain resulting from the use of the BSS network coloring mechanism;
- We investigated the impact of the proportional coexistence of legacy nodes with IEEE 802.11ax devices on network performance with the employed BSS coloring mechanism;
- We analyzed the impact of all three legacy standards, namely, IEEE 802.11ac, IEEE 802.11n, and IEEE 802.11a, using different MCS schemes, on the operation of the IEEE 802.11ax standard and the overall efficiency of the network;
- We examined the coexistence of IEEE 802.11ax and legacy stations with a variable share of devices (25% ax—75% legacy, 50% ax—50% legacy, 75% ax—25% legacy);
- We proposed the use of a frame-aggregation mechanism to optimize the operation of networks with overlapping BSSs and IEEE 802.11ax networks with legacy devices;
- We optimized the performance of mixed IEEE 802.11ax networks with various PHY and MAC parameters, such as OBSS_PD threshold, channel width, aggregation type and size, for both unsaturated and saturated channel conditions;
- We found that the performance of mixed IEEE 802.11ax networks is heavily dependent on the relative location of devices in the network, the presence of legacy nodes, the type of extension utilized, and the configuration of multiple mechanisms.

Section 2 provides a literature review of the IEEE 802.11ax standard related to the network coloring mechanism and/or coexistence with legacy stations. The legacy standard extensions are briefly described in Section 3. The next chapter focuses on the key mechanisms and techniques introduced to the IEEE 802.11ax standard extension. Section 5 is dedicated to the presentation of the results of the conducted research. The entire work is discussed in Section 6. Finally, Section 7 concludes the paper.

## 2. State of the Art

In [5] the authors, motivated by the significant increase in the number of devices in wireless networks in recent years, analyzed the IEEE 802.11ax standard and the mechanisms that were introduced aimed to improve the performance of networks with a large number of transmitting devices. Special attention was paid to techniques aimed at improving spectral efficiency, such as Dynamic Sensitivity Control (DSC) and network coloring. The research was based on a very dense network scenario, which included about 100 access points and over 1000 associated stations. It was confirmed that the use of a coloring mechanism increases the number of competitive transmissions, thereby increasing network performance. In the case of UL transmissions, a gradual increase in throughput was observed corresponding to the increasing number of IEEE 802.11ax stations. In the case of DL transmissions for a scenario with over 50% of legacy stations, a slight decrease in throughput was observed compared to a scenario with only legacy stations. It was also revealed that the increase in the number of competitive transmissions resulting from the use of the coloring mechanism is more favorable for stations located close to the access point, which achieve higher throughput. This phenomenon is due to the presence of numerous interferences with neighboring access points, which most affect the stations located at the edge of the network. The main conclusion derived from the research conducted by the authors is that the use of the coloring technique improves network performance only when there is a predominance of IEEE 802.11ax devices over legacy-type stations. Unfortunately, the research results only show the behavior of dense networks in which a very large number of nodes compete for access to the medium. The paper also omits the influence of the OBSS_PD parameter on network performance.

The authors of [6] proposed the COST algorithm (Control OBSS/PD Sensitivity Threshold), controlling the selection of the OBSS/PD threshold parameter and extending the basic mechanism of network coloring. Its assumption selects the mentioned parameter based on the level of interference in the network and the RSSI values of the stations connected to the access point. The aim of the algorithm is to select the OBSS/PD threshold in such a way as to minimize interference with overlapping networks, while maximizing the number of competing transmissions. COST was also proposed in response to the limitations of the existing DSC mechanism. The authors primarily wanted to maintain ongoing transmissions and provide more frequent access to the transmission medium for stations located at the edge of the network, as well as to ensure fairness for all stations wishing to initiate the transmission. According to the authors’ research, the proposed COST algorithm increased the network throughput. The best results were achieved for MCS values equal to 0. However, the use of higher modulations did not result in a significant increase in throughput due to the higher Signal to Interference and Noise Ratio (SINR) requirements. In the most favorable case, when using the COST mechanism, a 57% increase in throughput was observed for DL transmissions. The authors claim that the advantage of the implemented algorithm is the fact that, unlike the DSC mechanism, COST treats users located at the edge of the network more fairly, allowing for them to transmit more frequently. Regrettably, the COST algorithm increases the transmission opportunities for STAs (for all users within a BSS), resulting in a higher contention level. This limits the usefulness of this algorithm, especially when, in the BSS, a large number of stations are transmitting under saturation conditions.

Article [7] is also devoted to the analysis of BSS network performance using the coloring mechanism. The research scenario included 7 BSS networks consisting of one access point and 10 stations whose ranges overlap. The aim of the research was to find the optimal value of OBSS_PD threshold that would result in the highest gain in throughput collectively being achieved by all networks. The authors were aware that increasing the CCA threshold would also increase interference and the level of frame transmission errors, so they tried to minimize these by appropriately adjusting the aforementioned threshold. The research showed that increasing the value of the OBSS_PD threshold resulted in an increase in the total network throughput, but after exceeding a certain threshold, which, in this case, was −67 dBm, the throughput began to gradually decrease. This is due to the increase in the number of possible parallel transmissions and collisions. The authors observed the greatest increase in throughput for OBSS_PD values, equal to −72 dBm, which resulted in a throughput increase of 13.5%. Unfortunately, the authors present only one scenario with 7 BSSs and a fixed distance between the access points for which they determined the optimal size of the OBSS_PD parameter. They also assumed the only constant value of the load offered for each BSS. Their research, however, could narrow down the changes in the OBSS_PD parameter in our study to −78 dBm.

Article [8] is also dedicated to the analysis of the 1024-QAM modulation and network coloring mechanism in the IEEE 802.11ax network. The article presents a comparison to the IEEE 802.11ac standard extension. The main scenario that was studied involved a network consisting of three access points and several stations with the network coloring mechanism enabled and disabled. An increase in throughput of 14% was observed thanks to the use of the new 1024-QAM modulation, and an increase of up to 47% was observed in the case of using the network coloring mechanism. This work also has some limitations. The authors assumed that only 3 BSSs were located in line topology, constant value of OBSS_ED energy detection parameter, and only 1 MCS index = 11 for IEEE 802.11ax and the mobility of stations. It also assumes that network operation only occurs under saturation conditions.

The authors in article [9] conducted an analysis of the performance of Wi-Fi networks based on the IEEE 802.11ax extension when coexisting with IEEE 802.11n and IEEE 802.11ac stations. The research was carried out on a real network using COST devices and compared with the results obtained using ns-3 and Komandor simulators. The scenario was based on one access point and a different number of stations, ranging from 1 to 24. It was observed that, in the real network, as the number of clients increases, there is a significant decrease in throughput for IEEE 802.11ax, IEEE 802.11ac, and IEEE 802.11n stations, which is not observed in the simulation results. In the scenario that examined the impact of coexistence of legacy stations with the IEEE 802.11ax stations, a tendency was observed where, as the percentage of IEEE 802.11ax stations increased, UL transmission had an advantage over DL transmission in the network. Regrettably, an analysis of the network BSS coloring mechanism was left out of this work. The authors also concentrated only on scenarios with a single AP, a device that supports 802.11ax and a variable number of stations, including legacy IEEE 802.11ac and IEEE 802.11n devices.

Article [10], according to the authors, is the first work where an analytical model was proposed that establishes the optimal parameters of the coexisting networks at short distances using mechanisms introduced by the IEEE 802.11ax extension. It was observed that throughput degradation is directly related to the level of network interference. The authors analyzed the OBSS_PD parameter and the transmit power of the station to determine their optimal values. To maximize gain, when choosing transmit power, the authors considered parameters such as MCS, traffic direction, and transmission origin. The algorithm was designed to maximize the total throughput of multiple BSS networks broadcasting on the same channel. According to the research findings, in order to maintain similar interference levels for both simultaneously transmitting stations, the station should adjust its transmit power level accordingly. This allows for an appropriate OBSS_PD threshold to be set, which is closely related to interference. By applying an additional algorithm presented by the authors, and with the proper network configuration, a gain in throughput equal to 26% compared to the traditional network coloring mechanism can be achieved. This work also considers only one scenario with 7BSS and missed the the problem of coexistence with legacy stations. It also assumes a constant A-MPDU duration = 5 ms. All stations were configured with a full buffer, meaning that that the authors considered only saturation conditions.

Article [11] is devoted to an analysis of the network coloring mechanism with the use of traffic of different priorities. It was observed that the maximum increase in achieved throughput was 20%, obtained for the OBSS_PD parameter equal to −72 dBm. Additionally, the authors drew attention to the occurrence of a disturbance in the mechanism’s operation in the case of using a too-high OBSS_PD threshold value, which led to the inappropriate treatment of traffic class priorities. However, the authors were able to eliminate this phenomenon by using Request to Send (RTS)/Clear to Send (CTS) control frames. Regrettably, this paper only analyzed networks that adhere to the IEEE 802.11ax standard.

The authors in [12] propose a spatial reuse method for uplink, which can utilize BSS color and proximity information to improve the efficiency of carrier sensing and, thus, spatial reuse. A node receiving a preamble, and thus the BSS color and the proximity information, can figure out how far away the receiver of the ongoing traffic is located. This information is used to determine whether the node should aggressively start transmitting or defer its transmission to protect the ongoing transmission. The simulation results show that the proposed method outperforms existing methods in terms of throughput and fairness.

In [13], a new analytical framework for the 802.11ax MAC protocol is proposed. The authors use Markov-chain-based models to represent the behavior of the IEEE 802.11ax nodes, and both non-saturated traffic conditions. They also consider co-existence with the legacy nodes. Through both analysis and simulations, it is shown that the proposed model accurately evaluates the throughput and the delay performance under various network conditions. Unfortunately, the proposed model assumes OFDMA transmission and does not consider BSS network coloring.

In [14], the authors also evaluated the performance of the IEEE 802.11ac devices from two different perspectives: coexistence between Wi-Fi devices and coexistence between networks. They analyzed both the coexistence between heterogeneous (new and legacy) Wi-Fi devices (both stations and APs) and the inter-network interference (including old and new amendments). The research showed that poor network performance is inevitable, especially if networks are operating in close proximity. The authors also conclude that there are many unresolved problems, and additional mechanisms are needed to improve the coexistence of future Wi-Fi networks. Unfortunately, this work omits an analysis of the IEEE 802.11ax standard and functioning of the network coloring mechanism.

The authors in [15] investigate the impact of the aggregation level on the performance of real-time multimedia applications in IEEE 802.11n/ac WLANs. They present a novel end-to-end delay model for the unsaturated channel. They also propose a novel QoS-aware A-MPDU aggregation scheduler to obtain a better QoS performance with a larger capacity, lower latency and less packet loss. The validation of the model was confirmed using the NS-3 simulator. The proposed scheduler showed stronger adaptability in different scenarios.

In [16], the problem of throughput maximization based on optimized frame aggregation levels for IEEE 802.11 WLANs is presented. The authors propose a method to determine the optimal subframe sets for both A-MPDU and A-MSDU. The proposed scheme was validated through numerical calculations and Monte Carlo simulations. Although the computational complexity of the proposed scheme is larger than that of the conventional scheme, this leads to a significant throughput improvement.

To the best of our knowledge, the presented work is the first in the literature that discusses the impact of the BSS coloring mechanism on network efficiency where IEEE 802.11ax coexists with all possible types of legacy stations operating in a 5 GHz band in a typical dense office (Scenario 1) or residential scenario (Scenarios 2 and 3). In the following chapters, we present an in-depth description of the performance results of mixed networks with different topologies and configurations. We optimized the network performance using the OBSS_PD threshold parameter and frame-aggregation mechanism. The networks were evaluated under non-saturation and saturation conditions with a varying share of legacy stations.

## 3. The Legacy Devices of IEEE 802.11 Standard

At present, the IEEE 802.11 standard represents a broad family of extensions that have been appearing successively every few months. The first Wi-Fi standard was approved and published in 1997, but is completely obsolete at present. The ever-increasing requirements for the performance of wireless networks result in a constant need to introduce a number of changes, including the techniques and protocols used. Therefore, each new extension of the IEEE 802.11 standard, distinguished by successive letters of the alphabet, introduces improvements to the PHY and/or MAC layers.

As previously mentioned, the original IEEE 802.11 standard is no longer implemented due to the very low transmission rates, which reached a maximum of only 2 Mbps. It was designed to operate in the 2.4 GHz frequency band and presented three transmission techniques: Frequency Hopping Spread Spectrum (FHSS), Direct Sequence Spread Spectrum (DSSS), and Infra-Red (IR). The next extension was IEEE 802.11b, published in 1999, which introduced CCK modulation, allowing for transmission with 5.5 and 11 Mbps data rates. No changes were made to the transmission technique, and the devices still operated in the 2.4 GHz frequency band. However, this frequency band was already very crowded due to the presence of devices using other wireless standards, transmitting in the same frequency band [17]. In the same year, IEEE 802.11a was also approved, the first to operate in a much less congested 5 GHz frequency band, resulting in less interference but also a smaller radio range. A new transmission technique called OFDM was introduced, which theoretically allowed for a throughput of 54 Mbps.

Another important extension was standardized in 2003, IEEE 802.11g, which quickly replaced its predecessors due to the increasing demand for faster transmissions [18]. Like “b”, IEEE 802.11g is designed for operation in the 2.4 GHz band and, like “a”, uses OFDM for transmission. Working at these frequencies still involved numerous interferences, so communication was only possible in a few separated channels.

The publication of the IEEE 802.11n extension, which was named Wi-Fi 4 or the fourth generation of Wi-Fi, is considered a breakthrough in the standardization of wireless local area networks [19]. It introduced many changes to the PHY and MAC, significantly improving the range, reliability, and throughput. Devices compatible with IEEE 802.11n can operate in both 2.4 GHz and 5 GHz frequency bands. Additionally, the Multiple-Input–Multiple-Output (MIMO) technique was introduced, allowing for the simultaneous transmission and reception of multiple data streams using up to four antennas. Another change was the increase in channel width to 40 MHz. IEEE 802.11n also allowed for the use of a very short Guard Interval (GI) of 400ns, which enabled even higher throughput. All these enhancements enabled a sum of transmission rates ranging up to 600 Mbps, with a 4 × 4 MIMO configuration.

The next generation of Wi-Fi, Wi-Fi 5, was introduced in 2014 [20]. IEEE 802.11ac utilizes the 5 GHz frequency band and offers the possibility of using up to eight independent streams with an 8 × 8 MIMO configuration. This technique was further expanded by adding the ability to use Multi-User MIMO (MU-MIMO), which allows for independent transmission to several clients simultaneously. New channel widths were also added, which can be dynamically selected from 20, 40, 80, and 160 MHz, as well as the possibility of using a higher 256-QAM modulation. All of these improvements resulted in a noticeable real-world improvement in network performance, where the transmission rate at PHY can reach up to 6.93 Gbps.

## 4. IEEE 802.11ax

In 2014, the TGax began working on a new extension to the IEEE 802.11 standard, which was officially approved in 2020 [3]. This breakthrough was named Wi-Fi 6th generation. Previous standard extensions mainly focused on improving the performance of individual devices by using higher MCS indexes and a greater number of receiving and transmitting antennas, which are not sufficient under dense network conditions. A large number of devices have contributed to a significant increase in collisions and interference with neighboring BSS networks. The increasing number of hidden and exposed stations also affects network performance [21]. In addition, the current number of transmission channels has become insufficient, causing increasingly frequent appearances of Overlapping Basic Service Set (OBSS) networks and numerous collisions and interference [22]. IEEE 802.11ax, in response to the existing and worsening problems, has proposed many changes at the PHY and MAC level, as well as defining new mechanisms aiming to significantly improve the network spectral efficiency.

The IEEE 802.11ax extension is designed to work in both 2.4 GHz and 5 GHz frequency bands. A new transmission technique called Orthogonal Frequency Division Multiple Access (OFDMA) has been introduced. IEEE 802.11ax extends the capabilities of the MU-MIMO mechanism, which can now be applied to eight independent streams for both uplink (UL) and downlink (DL) transmissions. The TGax group designed several mechanisms to reduce energy consumption and improve spectral efficiency [23].

The IEEE 802.11ax extension introduced the use of 1024-QAM modulation, in which 10 bits are modulated to carry information. Its use potentially results in a 25% gain compared to the lower 256-QAM modulation, which uses eight bits to carry the signal. This expanded the range of the Modulation Coding Scheme (MCS) parameter by two additional values. MCS 10 and 11 are proposed, with respective codes efficiencies of 3/4 and 5/6. However, the use of such a high modulation is associated with high requirements for the Signal to Noise Ratio (SNR) value and a limited transmission range.

In addition, the symbol duration in OFDM technology was extended to 12.8 µs, which was four times shorter in previous standards. As a result, the spacing between the subcarriers reduced, and is now 78.125 kHz, allowing for the use of 256 subcarriers. To minimize the impact of interference, which is a crucial aspect when using the highest order modulation, it is possible to use three different GI values, which can be 3.2 µs, 1.6 µs, or 0.8 µs. Using the smallest value minimizes the additional transmission overhead, but then the GI is the least effective in countering the effects of signal propagation through multiple paths. Similarly to the previous IEEE 802.11ac standard, it is possible to use four channel widths—20 MHz, 40 MHz, 80 MHz, and 160 MHz—as well as eight independent streams in MIMO technology.

Several headers were also added to IEEE 802.11 frames in Wi-Fi 6. The first is High-Efficiency Single User (HE SU), designed for transmission to single users. Its counterpart, for transmitting to multiple clients, is High-Efficiency Multi-User (HE MU). High-Efficiency Extended-Range Single User (HE ER SU) is designed for transmitting to single users over long distances, which increases their transmission power by 3 dBm and allows for the use of low-value modulations. The last type is High-Efficiency Trigger-Based (HE TB), which is broadcast to all clients and is used for Uplink transmission.

IEEE 802.11ax also introduces a 2-MHz-only operation mode, which allows for the use of all available features of the latest extension, but only within a single 20 MHz channel. This solution is useful for Internet of Things (IoT) devices, e.g., sensors that have a cheaper and less complex physical architecture but can still use new mechanisms [24,25].

Wi-Fi 6 devices, through their ability to communicate in both frequency bands, are compatible with older wireless network standard extensions. In addition, the IEEE 802.11ax extension allows for the use of outdated security mechanisms that do not meet the current requirements but still allow for cooperation with legacy stations.

Wi-Fi 6 also offers several changes in the operation of the MAC layer. One of the changes is the increased maximum size of the aggregated frame, which can reach up to 11,398 bytes for A-MSDU, and up to 8,388,607 bytes for A-MPDU. A new type of aggregation has also been proposed in the Multi-Traffic Identifier Aggregated MAC Protocol Data Unit (Multi-TID AMPDU), which aims to group frames with different traffic class identifiers, which was previously impossible. This approach allows for increased network efficiency by minimizing additional control frame communication, which occupied a significant portion of teh available bandwidth [3].

The latest extension also proposes many completely new mechanisms that contribute to the increased spatial utilization of the radio channel. Of particular importance is the network-coloring mechanism, multi-user techniques, and the newly introduced Orthogonal Frequency-Division Multiple Access (OFDMA) transmission technique, which are described in the following subsections.

### 4.1. BSS Network Coloring Mechanism

The key mechanism offered by the IEEE 802.11ax standard that significantly improves spectral efficiency is network coloring. This was first introduced in the IEEE 802.11ah and partially based on the Partial AID (PAID) algorithm presented in the IEEE 802.11ac.

**Figure 1:**
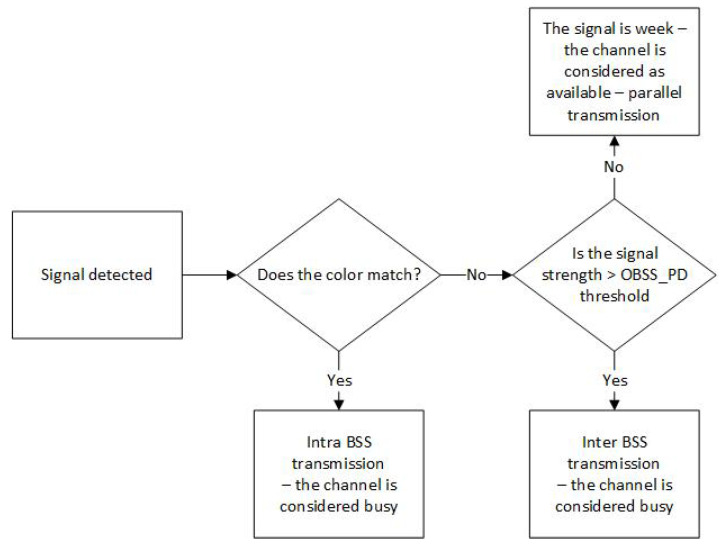
The BSS coloring algorithm in the IEEE 802.11ax standard.

Its operation is based on the labeling of each transmission with an additional identifier, which is the color, allowing for a station to distinguish the origin of the frame and process only those sent by devices belonging to the same BSS network (transmissions with the same color). To enable the transmission of additional information, a 6-bit SIG-A field has been defined, which is located in the preamble of the PHY. In the MAC sublayer, the color is carried by management frames. Six bits allowed for the use of 63 colors, and the value 0 indicates the disabled mechanism. The color is chosen by the access point, which can have a predefined value or choose it independently by analyzing the available colors in the network. However, simply filtering frames based on the identifier and rejecting those coming from a foreign BSS network only slightly improves network efficiency. Therefore, an additional carrier detection threshold was defined in the mechanism, which is the Overlapping BSS Preamble Detection (OBSS_PD) threshold [10,11,12]. This is only used by the access point when a frame from another BSS network is detected. Based on this, the decision is made to start or stop the next parallel transmission. The range of OBSS_PD threshold values directly results from the maximum transmission power set in the device according to the following formula:

(1) $$\begin{array}{c} OBSS_{PD}\leq max\left(OBSS_{PDmin},min\left(OBSS_{PDmax},OBSS_{PDmin}+\left(TX_{PWRref}-TX_{PWR}\right)\right)\right) \end{array}$$

where $OBSS_{PDmin}=-82$ dBm, $OBSS_{PDmax}=-62$ dBm, $TX_{PWRref}=21$ dBm, and $TX_{PWR}$ denote the station’s transmit power in dBm [3]. This control was introduced to prevent traffic degradation due to excessive collision growth in the network. An IEEE 802.11ax station or access point receiving a frame analyzes the color contained in the preamble. If it matches its own, the transmission is recognized as Intra-BSS, which means it originates from the same network. Then, another station that also wants to start a transmission considers the channel occupied. However, if the received frame has a different color, the transmission is recognized as Inter-BSS, which means it comes from a foreign network, and the station starts analyzing the received signal’s power. The received signal is compared to the OBSS_PD threshold. If the RSSI is less than OBSS_PD, the station considers the channel free and can start a parallel transmission. Otherwise, the channel is deemed occupied, and the station cannot start transmitting the frame due to the high probability of collision. The detailed operation of the algorithm is presented in the diagram (see Figure 1).

In addition to increases in spectral efficiency, the network coloring mechanism aims to reduce energy consumption by quickly identifying the origin of the frames.

### 4.2. OFDMA and MU-MIMO

A key change introduced by the IEEE 802.11ax standard, which significantly improves spectral efficiency, is the Orthogonal Frequency Division Multiple Access (OFDMA) transmission technique [26]. This is a kind of extension of the OFDM technique, which is intended for multiple users. The channel resources are divided into units called Resource Units (RUs), which allow for multiple clients to transmit data simultaneously. Each client is allocated a separate RU, which can be composed of different numbers of subcarriers. Using a 20 MHz channel, the access point can sequentially allocate 26, 52, 106, or 242 subcarriers to each station. The OFDMA technique is a good solution for transmitting many small-sized frames, as it relieves the MAC layer, thereby reducing delays and increasing network efficiency [27].

MU-MIMO is another technique aiming to achieve simultaneous communication with multiple clients. It was introduced in the IEEE 802.11ac, but at that time its application was possible only for DL transmission and a maximum of four clients simultaneously. MIMO technique allows for the use of up to eight streams, but client devices rarely have more than two antennas, which results in unused potential in the access point. MU-MIMO solves this problem by communicating with multiple clients at the same time and using all available streams. This mechanism is managed by the access point, which transmits a control frame containing synchronization information and information about the group of clients that can transmit at the same time. IEEE 802.11ax allows for the simultaneous transmission of eight clients for both UL and DL communication. However, MU-MIMO operation requires the exchange of Transmit Beamforming (TxBF) frames, which add additional overhead, especially when transmitting small messages [28].

## 5. Performance Evaluation

All simulation studies described in the paper were performed using the Network Simulator (NS) version 3.36. NS-3 is an event-driven simulator written in C++, primarily intended for educational purposes, which is available free of charge under the GNU GPLv2 license [29]. Version 3.36 introduced many improvements in the operation of the IEEE 802.11ax wireless network, which allowed for more reliable results.

All research scenarios presented in this work are based on client–server communication. In the client stations, an application acting as a source was installed, generating a constant bit rate (CBR) traffic. In scenarios based on communication with multiple stations, traffic-generating applications were launched with a random delay ranging from 0 to 1 s. The warmup time in simulator was set to 5 s. To make the simulation environment as close to reality as possible, the Friis loss propagation model was used. Each of the experiments were conducted and the results with and without the BSS coloring mechanism were compared. Table 1 presents the most important values of PHY, MAC, and higher-layer parameters that are common to all simulations. In all figures, the error in each simulation point for the 95% confidence interval did not exceed ±2%.

### 5.1. Scenario 1—Analysis of BSS Coloring Mechanism

The first scenario examined the impact of the number of coexisting BSS networks within range on the throughput gain resulting from the use of the network coloring mechanism introduced by the IEEE 802.11ax. Throughput was analyzed for different values of the OBSS_PD parameter, with aggregation enabled and disabled. Multiple simulations were executed for different distances, D1, between networks. Each BSS has one associated station located two meters from its access point. Additional parameters used in this scenario are listed in Table 2. All results were collected for uplink transmission and under network saturation conditions to better observe the occurring phenomena. Additionally, to test the network coloring mechanism, all BSS networks used the same channel number, 36, for communication. The results presented in the following subsections show the throughput that was achieved for all networks.

#### 5.1.1. Two BSS Network Topology

**Figure 2:**
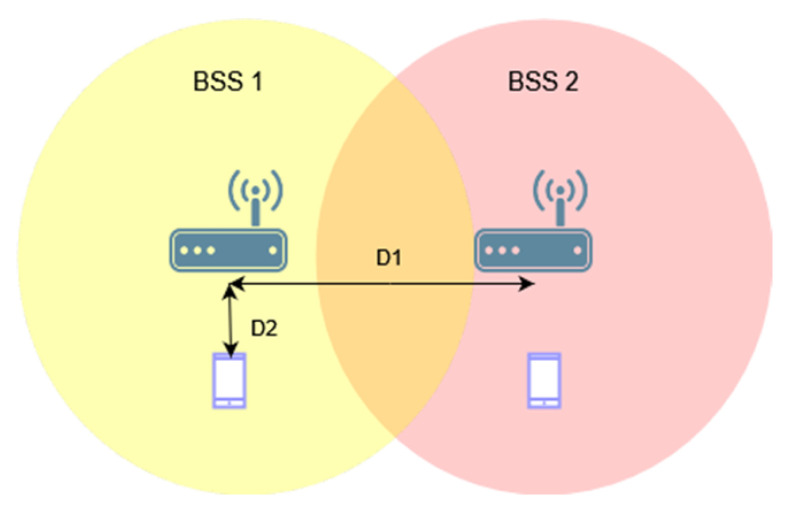
Two BSS network topology.

Figure 2 shows the first analyzed topology, consisting of two access points and two stations. By using different distances, D1, between access points, the area of mutual radio coverage is changed, which can affect the number of collisions in the network.

**Figure 3:**
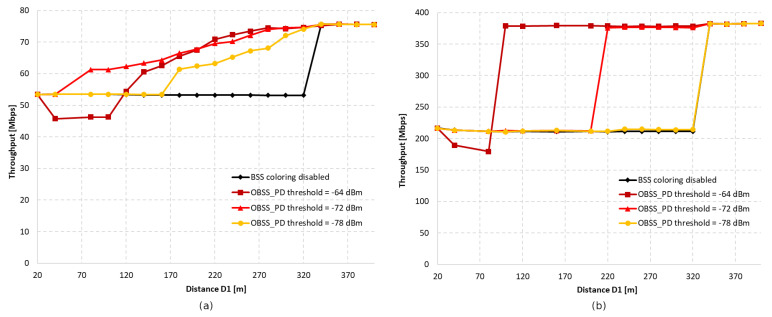
Throughput as a function of D1 distance for different OBSS_PD threshold values and two BSS: (**a**) aggregation disabled and (**b**) aggregation enabled.

Figure 3a shows the throughput as a function of the distance between access points with aggregation disabled for OBSS_PD threshold values of −64 dBm, −72 dBm, and −78 dBm, respectively. We assumed that we would analyze only three selected OBSS_PD values from the possible range −62–−82 dBm. Based on our previous research [11], as well as the conclusions drawn in [7], we noticed that the largest changes in the obtained throughput occur in the range −64–−78 dBm. Therefore, three values were selected, which differed from each other by a constant value of 6 dBm. The range of each BSS can be observed on the black line, which represents the change in throughput when the coloring mechanism is disabled. It was observed that both networks are completely isolated when access points are deployed at a distance of 340 m, which means that the range of each AP is about 170 m. In the absence of network overlap, its maximum throughput was less than 40 Mbps, whereas in a situation where stations were forced to share the same channel, the throughput decreased by almost half. It was observed that, with an increase in the distance of D1, a greater gain can be obtained the application of the coloring mechanism. This is primarily due to the fact that as access points move away from each other, the signal strength received by stations decreases, and they compete for access to the channel with another station that is trying to transmit simultaneously. This provides more transmission opportunities, which, along with greater network isolation, becomes more resistant to collisions and interference. Additionally, by using lower OBSS_PD threshold values, a greater increase in throughput was achieved. This is due to the coloring mechanism, which allows for parallel transmission when a frame from a foreign BSS network is detected with lower power than the configured OBSS_PD threshold parameter. Therefore, if access points are in close proximity, the use of a low threshold will result in the constant rejection of parallel transmission, negating any possible gain. Therefore, the observed increase in throughput is for a threshold value of −78 dBm at a distance D1 of only 180 m. However, a high threshold does not always lead to the greatest gain, as was observed for the OBSS_PD threshold value of −64 dBm. The close proximity of neighboring BSS stations and frequent permission for parallel transmission by the mechanism caused numerous collisions that led to a significant decrease in throughput, which, in the worst case, dropped by 15%. The detailed analysis shows that the use of a −64 dBm threshold leads to a number of collisions, which prevent both the correct decoding of frames and the proper setting of the NAV allocation vector for WLAN devices. If the distance D1 is further reduced, the NAV is correctly set based on the reception of frame headers from all BSSs, and the simultaneous transmissions from BSSs located close to each other are avoided (the throughput is split evenly between the two BSS areas). Therefore, the optimal threshold value for the given scenario was −72 dBm, as it provided a gain in the achieved throughput for the widest range of D1 distances.

The research was also repeated using an A-MPDU aggregation of 65,535 bytes, the results of which are presented in Figure 3b. The main observed difference compared to the lack of aggregation is the sudden gain in achieved throughput when the coloring mechanism is used. The reason for this behavior may be that, by using frame aggregation, the overhead of control frames and the number of channel access attempts reduced, which contributed to the reduced number of collisions. Similar to the lack of aggregation, a decrease in throughput was observed for an OBSS_PD threshold value of −64 dBm in the case of close network deployment, which also reached a maximum of 15%. However, this phenomenon lasted for a shorter period than in the previous scenario, and at a distance value of D1 equal to 100 m, a significant gain from the use of the coloring mechanism was observed. When using aggregation, an OBSS_PD threshold parameter value of −64 dBm provided a gain for the largest range of distances between access points.

#### 5.1.2. Three BSS Network Topology

**Figure 4:**
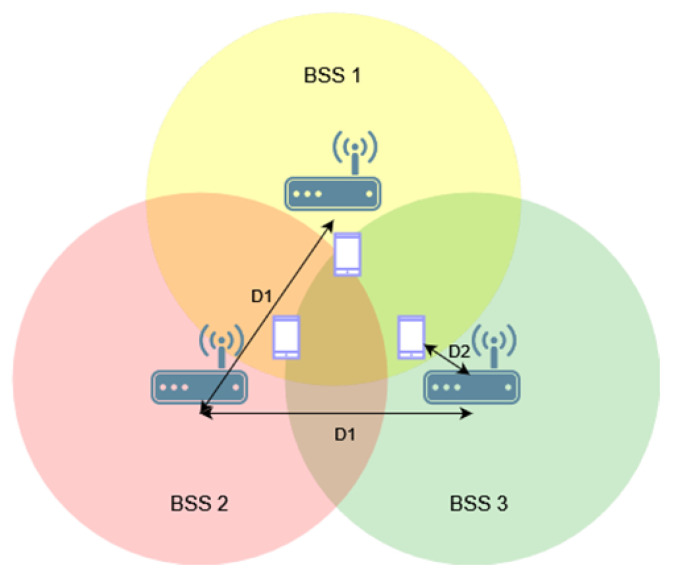
Three BSS network topology.

Next, the same study was conducted for three networks arranged in a triangle topology, as shown in Figure 4. This resulted in the creation of an area that all or at least two access points could cover with their range.

**Figure 5:**
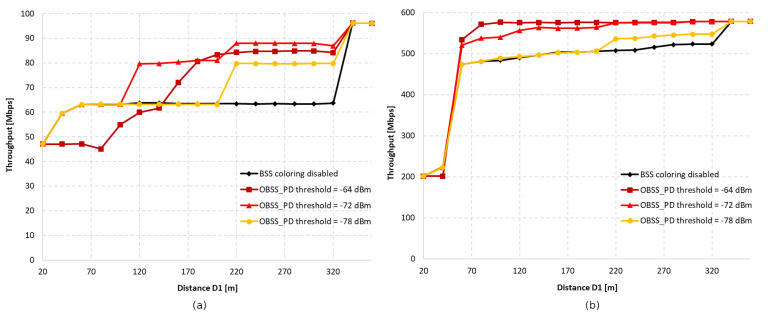
Throughput as a function of D1 distance for different OBSS_PD threshold values and three BSS: (**a**) aggregation disabled and (**b**) aggregation enabled.

Similarly to before, the impact of using the coloring mechanism and different OBSS_PD threshold values on throughput in networks without configured aggregation was first examined, as presented in Figure 5a. Two clear increases in throughput were observed when using OBSS_PD values of −64 dBm and −72 dBm. The smaller gain within a certain range of distances may be due to insufficient spacing between access points, which determines the size of the area where the coverage of the three networks overlaps. The previously observed sudden drop in throughput is even more pronounced in the given scenario, and was reduced by a maximum of 28%. For a threshold value of −64 dBm, the gain resulting from parallel transmission is visible only when the networks are separated by a distance of 160 m. This time, the most favorable OBSS_PD threshold value was also −72 dBm, which provided the fastest and greatest gain from using the new mechanism.

Figure 5b shows the throughput achieved when aggregation is used. It was observed that enabling aggregation results in lower throughput loss in situations where the coverage areas of networks without the configured coloring mechanism overlap. This behavior may indicate that, in the network, a large portion of transmissions are completed without collisions, even when multiple networks are transmitted in the same channel. Aggregation, therefore, further highlights their importance, resulting in smaller throughput losses. For small distances between access points, reduced throughput is also noticeable, but is much smaller than when aggregation is not enabled. The OBSS_PD threshold equal to −62 dBm, as for the two BSS networks, provided the highest gain in achieved throughput.

#### 5.1.3. Four BSS Network Topology

**Figure 6:**
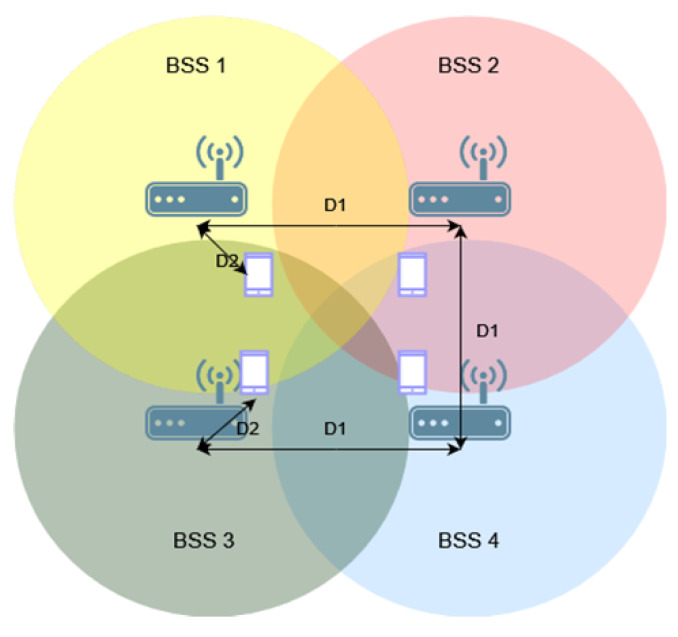
Four BSS network topologies.

The study was also repeated for four coexisting networks in the same channel, in the configuration presented in Figure 6.

**Figure 7:**
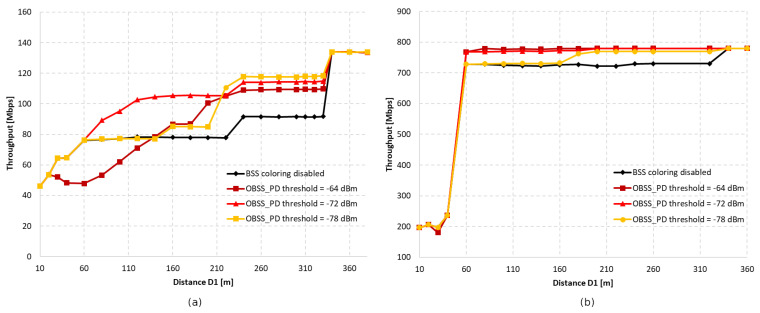
Throughput as a function of D1 distance for different OBSS_PD threshold values and four BSS: (**a**) aggregation disabled and (**b**) aggregation enabled.

In Figure 7a, the results of the throughput achieved by all networks without aggregation are presented. As in the previous case, the increase in throughput for certain distances was step-like. The increase in throughput for the configuration with the coloring mechanism that was turned off at the range of 220–240 m represents a situation where the networks on the diagonals are already isolated from each other and each has only two adjacent networks within range. As with the previous configurations, the gain is first noticeable when a threshold of −72 dBm is applied. Additionally, applying a threshold that is too high for short distances also results in a significant decrease in throughput, reaching up to −37% for a distance of 60 m. However, unlike in previous scenarios, although the OBSS_PD threshold value of −72 dBm showed the fastest gain, it was the value of −78 dBm that allowed for the highest throughput at the appropriate distance D1 (220–330 m). This means that the number of parallel transmissions allowed by such a threshold caused the fewest collisions in the network.

The use of aggregation for a topology consisting of four networks further amplified the phenomenon of a sudden increase in throughput at the range of from 40 to 60 m for the network-coloring mechanism, both enabled and disabled—see Figure 7b. This is due to the value of the CCA threshold, which, for a D1 value of less than 60 m, too often classifies the channel as busy. As with the three coexisting networks in the same channel, the greatest gain was observed for OBSS_PD thresholds equal to −64 dBm and −72 dBm.

#### 5.1.4. Seven BSS Network Topology

**Figure 8:**
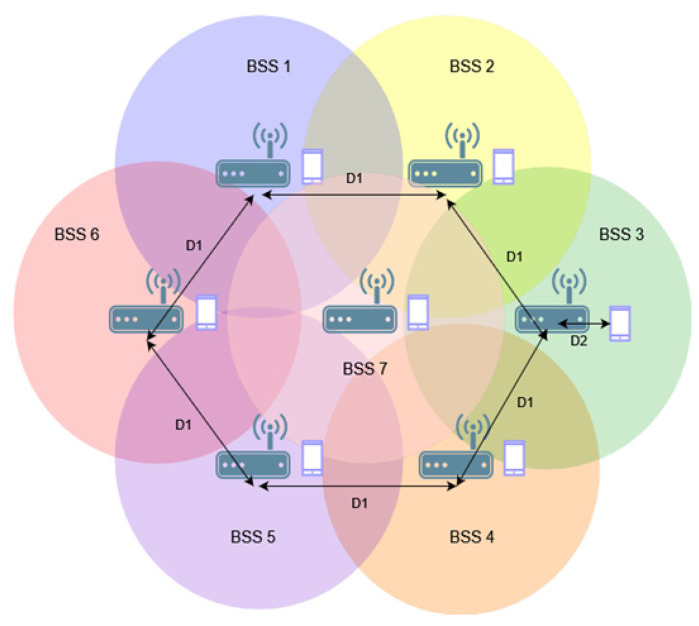
Seven BSS network topology.

The last study in this series was conducted for seven BSS networks, whose access points were located at the vertices of a regular hexagon with an additional network in the middle, as shown in Figure 8.

**Figure 9:**
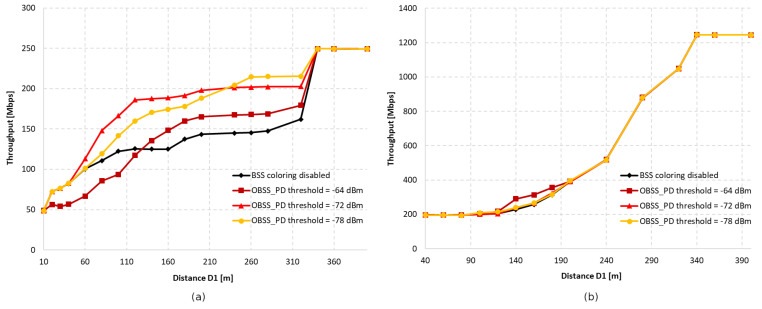
Throughput as a function of D1 distance for different OBSS_PD threshold values and seven BSS: (**a**) aggregation disabled and (**b**) aggregation enabled.

The complexity of the network caused the throughput in the absence of the coloring mechanism to become increasingly dependent on the distance between access points—see Figure 9a. Setting the OBSS_PD threshold parameter to the highest value resulted in a decrease in throughput of up to 33%. Similar to the case of four BSS networks, the largest gain was observed with a low threshold set at −78 dBm. The reason for this is the increase in the number of collisions with a larger number of transmitting stations, which is limited by the lower threshold.

According to the conducted research, however, the use of aggregation in the case of multiple coexisting networks with network coloring does not result in increased throughput—Figure 9b. This may be due to the fact that denser networks experience more interference and collisions, which aggregation further amplifies because, in case of errors, larger amounts of bits are lost and frames should be retransmitted.

#### 5.1.5. Summary

**Figure 10:**
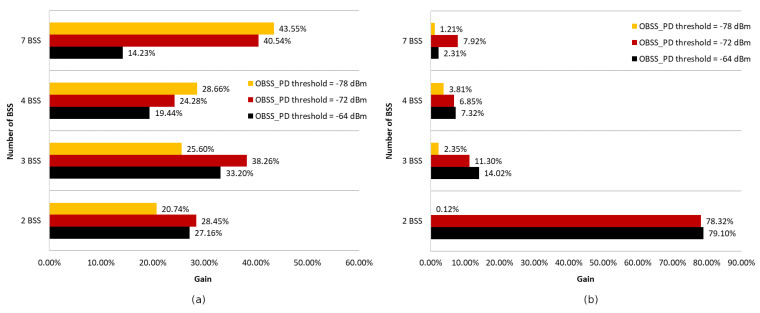
The average percentage increase in throughput using a coloring mechanism for different BSS and OBSS_PD threshold values: (**a**) aggregation disabled and (**b**) aggregation enabled.

The charts presented in Figure 10 summarize all four of the scenarios described above. Figure 10a shows the average gain in achievable throughput resulting from the use of a network coloring mechanism for each OBSS_PD threshold value in the absence of aggregation, while Figure 10b shows the data for enabled aggregation. The average was calculated for the distance range between access points from 220 to 320 m.

The simulations showed that the operation of the network coloring mechanism strongly depends on the level of interference, the number of collisions, and the location of wireless devices. In all scenarios without frame aggregation, using a high threshold value such as −64 dBm results in the smallest gain. Additionally, it was observed that use at small distances between networks causes a decrease in throughput of up to 30%. In topologies consisting of two or three BSS networks, the most favorable was the use of a threshold of −72 dBm, while for four and seven networks, a threshold of −78 dBm yielded the highest throughput gains. Thus, there is a dependence: the optimal OBSS_PD threshold value decreases as the number of networks operating in the same channel increases. This is mainly due to the fact that an increasing number of coexisting networks also causes an increase in the bit error rate and collisions, as well as more frequent occurrences of hidden stations. A high OBSS_PD threshold parameter, on the other hand, increases the possibility of coexisting parallel transmissions, which, under inappropriate conditions, contribute to the emergence of additional collisions, which collectively reduce throughput.

An interesting observation is that with an increasing number of overlapping networks, there is an increasing tendency for a percentage gain in achievable throughput. The reason for this is the operation of the network coloring mechanism, which requires the presence of interference and collisions that it can eliminate, which increase with the increase in coexisting networks.

According to the chart presented in Figure 10b, the simultaneous use of aggregation and network coloring mechanisms resulted in a huge increase in throughput, reaching 79%, but only in the case of two coexisting networks. Increasing the number of access points causes a decreasing gain, which, for the three BSS networks was only 14% and, for four and seven networks, was only 7%. It can be concluded that the use of aggregation negatively affects the operation of the network coloring mechanism in the case of high OBSS interference. Grouping frames generally significantly increases the throughput by limiting the exchange of control frames; however, in the case of collisions in the network, much larger data frames are lost, which can lead to smaller gains when using the network coloring mechanism.

**Figure 11:**
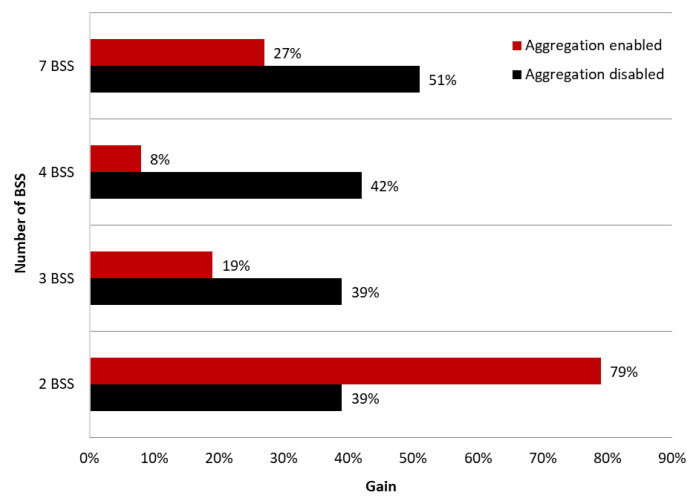
The maximum percentage increase in throughput using a coloring mechanism for different numbers of BSS and with the aggregation mechanism disabled or enabled.

The Figure 11 also shows the maximum gain achieved using a coloring mechanism for different numbers of BSS areas with aggregation disabled and enabled. It is notable that regardless of the number of BSS areas with aggregation disabled, a similar gain, ranging from 39% (two BSS and three BSS) to 51% (seven BSS), can be achieved. Enabling aggregation results in significant differences in network performance. The smallest gain achieved, which is only 8%, was for four BSS, while the largest gain, which was as much as 79%, was for two BSS.

Scenario 1 is summarized in Table 3. This includes mean throughput values in Mbps, averaged from the throughput values for all analyzed distances D1, with aggregation and BSS coloring mechanism disabled and enabled (assuming different OBSS_PD parameter values).

### 5.2. Scenario 2—Proportional Coexistence of IEEE 802.11ax and Legacy Stations

The next scenario investigates the impact of the coexistence of legacy stations with IEEE 802.11ax devices on network performance with the coloring mechanism disabled and enabled. The distance between access points and the OBSS_PD threshold value were selected based on the results of the previous scenario, so that a noticeable gain in throughput would be observed. Simulations were conducted for different channel widths and aggregation configurations.

**Figure 12:**
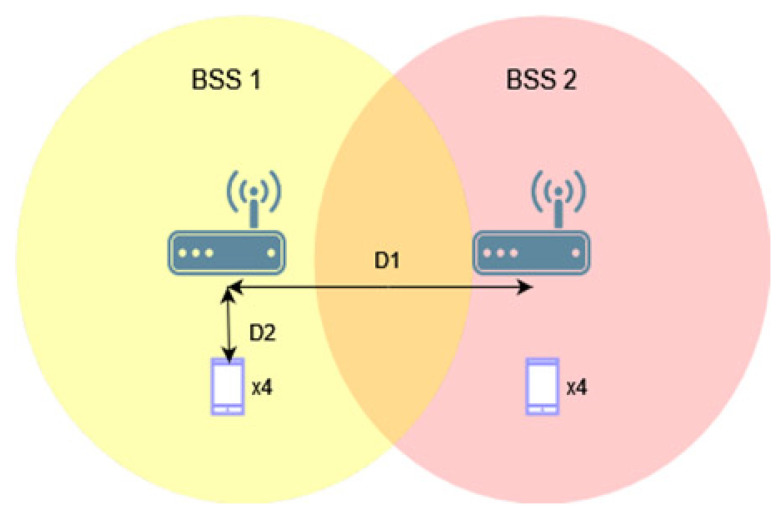
Network topology for mixed IEEE 802.11ax networks.

The network topology consisted of two access points, four IEEE 802.11ax, and four legacy stations, as shown in Figure 12. The scenario investigates three standard extensions, namely IEEE 802.11ac/n/a. Table 4 includes additional assumed network parameters.

#### 5.2.1. IEEE 802.11ax + IEEE 802.11ac

**Figure 13:**
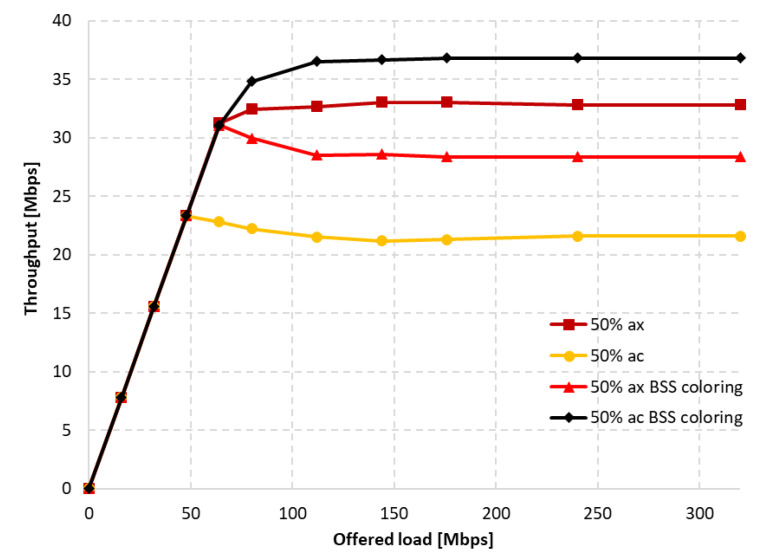
Throughput vs. offered load for mixed ax (50%) and ac (50%) networks without aggregation.

Firstly, the coexistence of IEEE 802.11ac legacy stations was analyzed using a channel width of 80 MHz. Three aggregation configurations were assumed: disabled, default, and maximum. Figure 13 illustrates the throughput achieved by both networks, with and without aggregation, as well as with and without the coloring mechanism. Throughput was presented separately for each extension, while gradually increasing the offered traffic until the network became saturated. It was observed that, with low network traffic, all stations achieved similar throughput. However, under saturation conditions, some stations obtained a significant advantage. When the network coloring is disabled, in saturated conditions, IEEE 802.11ax stations achieved a 36% higher throughput compared to legacy stations. Although enabling the coloring mechanism resulted in a total network throughput increase of about 20%, this was mainly observed for IEEE 802.11ac stations. In the analysed scenario, IEEE 802.11ax stations achieved an even lower throughput than with the coloring mechanism disabled. This unexpected phenomenon may indicate that stations that do not implement the coloring mechanism do not follow the new rules in the network, allowing for more frequent transmissions.

**Figure 14:**
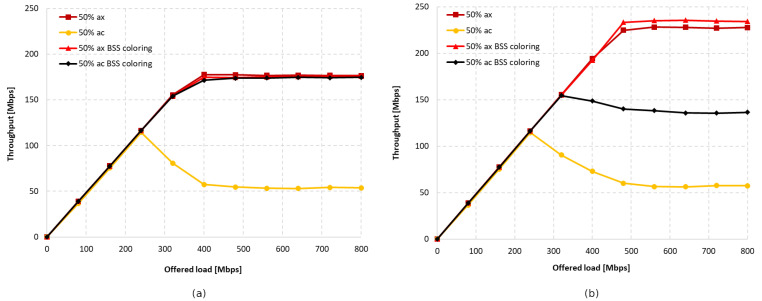
Throughput vs. offered load for mixed ax (50%) and ac (50%) networks: (**a**) default aggregation and (**b**) maximum aggregation.

Figure 14a shows the throughput achieved by individual stations when using the default aggregation for Best Effort traffic, which is A-MPDU with a value of 65,535 bytes, for both legacy and IEEE 802.11ax clients. In this case, the gain resulting from the use of the network coloring mechanism reached about 53%. Additionally, the IEEE 802.11ax stations no longer experienced a drop in throughput when using color in transmissions, and remained at the same level. However, the biggest gain compared to the mechanism being turned off was observed for legacy stations, which are now striving to achieve the same throughput as IEEE 802.11ax stations.

The maximum A-MSDU and A-MPDU aggregation values were then applied for each standard, which were 7935 bytes and 6,500,631 bytes for IEEE 802.11ax stations, and 7935 bytes and 65,535 bytes for IEEE 802.11ac devices. The results of the throughput achieved with and without the network coloring mechanism are shown in Figure 14b. The percentage gain in achieved throughput for both networks resulting from the use of the network coloring mechanism reached about 30%. However, there is a noticeable change in the proportion of achieved throughputs by IEEE 802.11ax and legacy stations, as both are treated in a more similar way when the coloring mechanism is disabled, and newer extensions under network saturation conditions obtain a greater portion of the available transmission opportunities. Nevertheless, the IEEE 802.11ax stations still achieve a lower percentage gain resulting from the use of color. Aggregation, therefore, caused the phenomenon of cutting off IEEE 802.11ax station traffic in the case where there was network saturation to be eliminated. This is due to the reduction in the number of contention attempts for the channel and the IEEE 802.11ax stations being able to transmit more data.

**Figure 15:**
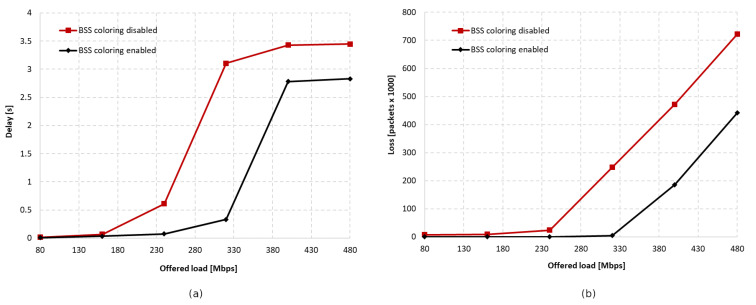
Mean packet delay (**a**) and packet loss (**b**) vs offered load for mixed ax (50%) and ac (50%) networks.

In addition, for the scenario where maximum aggregation was used, network delay and packet loss level were determined. Figure 15a shows the total network delay depending on the offered traffic, while Figure 15b shows the total packet loss, and the data that were collected throughout the simulation period. The use of the coloring mechanism has a positive impact on both metrics, reducing their value. By using the coloring mechanism, it is possible to transmit a larger amount of traffic with a small delay (320 Mbps) compared to when not using the mechanism (240 Mbps).

The simulations were also repeated for a 20 MHz channel width with disabled and maximum aggregations (we do not present figures due to space limitations). Similarly to the 80 MHz width, the lack of aggregation also resulted in a decrease in throughput for IEEE 802.11ax stations during gradual network saturation, and an increase for IEEE 802.11ac stations. In this case, however, legacy clients did not achieve higher throughput than IEEE 802.11ax clients, and the overall gain resulting from the use of coloring mechanism was 8%. The network configuration with maximum aggregation slightly improved the throughput for IEEE 802.11ax stations. The use of the coloring mechanism enabled throughput to be increased by up to 55%, but legacy stations still benefited the most.

#### 5.2.2. IEEE 802.11ax + IEEE 802.11n

Research based on the same topology was conducted for an even older extension, which is IEEE 802.11n, both with the highest channel width of 40 MHz, offered by the legacy extension and the basic 20 MHz.

**Figure 16:**
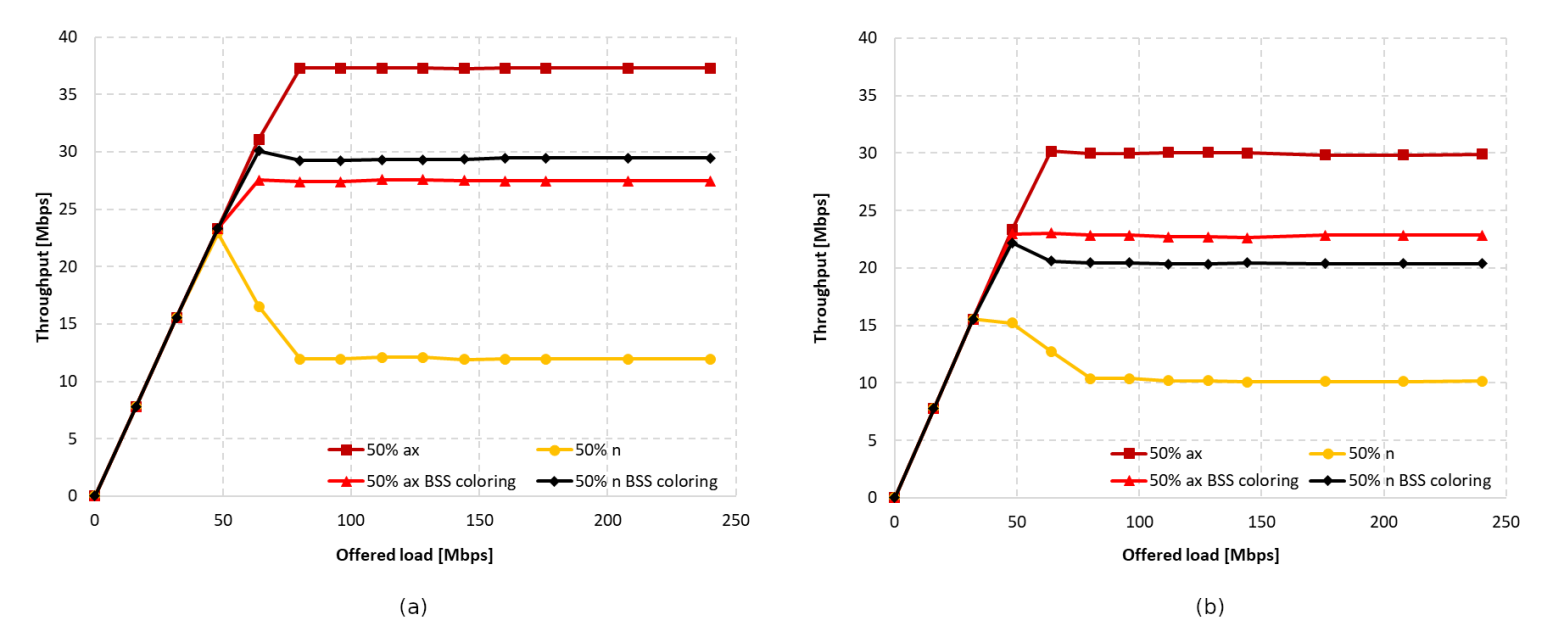
Throughput vs. offered load for mixed ax (50%) and n (50%) networks without aggregation: (**a**) 40MHz channel and (**b**) 20 MHz channel.

**Figure 17:**
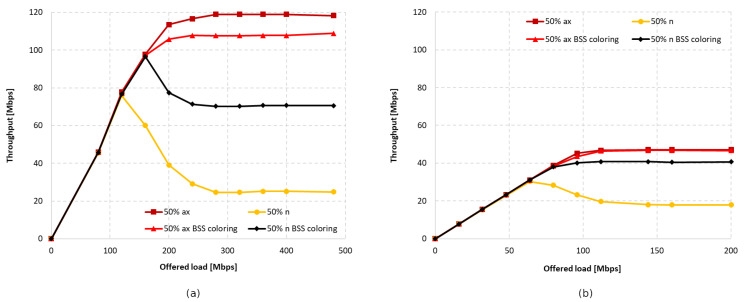
Throughput vs. offered load for mixed ax (50%) and n (50%) networks with maximum aggregation: (**a**) 40MHz channel and (**b**) 20 MHz channel.

Figure 16a,b shows the throughput graphs for disabled aggregation with channel widths of 40 MHz and 20 MHz, respectively. Figure 17a,b shows the achieved throughput when using the maximum possible aggregation for the standard, which was 65,535 bytes for A-MPDU and 7935 bytes for A-MSDU in this case.

As with the IEEE 802.11ac extension, the wide 40 MHz channel and lack of aggregation resulted in legacy clients dominating, as presented in Figure 16a. The total throughput of both networks increased by 15% with the use of the coloring mechanism, but this increase occurred only for the IEEE 802.11n. Using the basic channel width only resulted in a slightly higher throughput for IEEE 802.11ax devices, but this was still lower than when the coloring mechanism is turned off. This can be seen in Figure 16b. The total gain when using a 20 MHz channel width was about 8%, similar to when an IEEE 802.11ac station was present. Aggregation improved the balance between throughput for legacy and IEEE 802.11ax stations; however, as before, the gain was mainly observed for the older extension. The A-MPDU and A-MSDU configurations resulted in a total network throughput increase of about 25% for a 40 MHz channel width and 35% for a basic 20 MHz channel width (see Figure 17a,b).

#### 5.2.3. IEEE 802.11ax + IEEE 802.11a

The last examined IEEE 802.11 extension that was IEEE 802.11a, which is the oldest extension that allows for devices to operate in the 5 GHz frequency band. Additionally, it does not allow for frame aggregation or the use of wider channels. Therefore, the research was conducted for a 20 MHz channel width.

**Figure 18:**
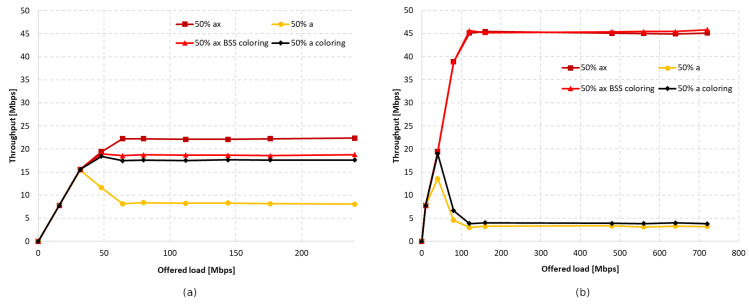
Throughput vs. offered load for mixed ax (50%) and a (50%) networks: (**a**) without aggregation and (**b**) maximum aggregation.

In Figure 18a, the throughput as a function of the offered traffic is presented for both standards without aggregation. Figure 18b, on the other hand, shows the throughput that was achieved with the maximum aggregation for IEEE 802.11ax stations. With the coloring mechanism turned off and no aggregation, the throughput for IEEE 802.11a station significantly decreased as the network became saturated. Enabling aggregation caused the same phenomenon as in the presence of IEEE 802.11ac/n stations, i.e., the equalization of throughput for all clients and a sharp gain for legacy clients. In the case of coexistence in the network of legacy stations, the simultaneous activation of aggregation and coloring mechanism for IEEE 802.11ax clients resulted in a negligible gain in achieved throughput, which amounted to 2%.

#### 5.2.4. Summary

**Figure 19:**
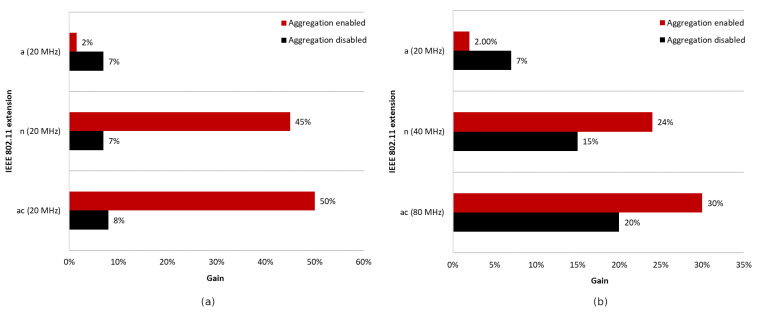
The average percentage increase in throughput using a coloring mechanism for different IEEE 802.11 standard extensions with aggregation enabled and disabled: (**a**) 20 MHz channel and (**b**) highest available channel width for a given extension.

The charts presented in Figure 19 summarize the studies conducted in the second scenario. They illustrate the percentage gain obtained from using the coloring mechanism in a network with aggregation enabled and disabled in the presence of three legacy standards, namely IEEE 802.11ac/n/a. The graphs are divided into two cases. Figure 19a shows the percentage gain for the basic channel width of 20 MHz, while Figure 19b shows the highest available channel width for a given legacy extension.

It was observed that, using a channel width of 20 MHz and disabling aggregation, the total network gain resulting from the use of the coloring mechanism is similar for all legacy standards, at approximately 7–8%. Wider channels provided even greater gains, of about 20% for 80 MHz and 15% for 40 MHz. This trend may be due to the presence of a larger number of collisions and interference in the case of wider channels, which causes a greater decrease in throughput that the coloring mechanism is able to compensate. Enabling aggregation, on the other hand, caused a gradual decrease in the percentage gain for all used channel widths. In the worst case, where IEEE 802.11ax and IEEE 802.11a stations coexist, almost no gain from the use of the coloring mechanism is observed, which is an unfavorable phenomenon. Additionally, with the coloring mechanism enabled and the presence of legacy stations, all stations achieve very similar throughput, which is unfavorable for IEEE 802.11ax clients, who lose throughput at the expense of older stations. This phenomenon seems to deepen with the use of larger channel widths and the reduction in the maximum value of aggregated frames. In the extreme case, which is a configuration with disabled aggregation and a wide 80 MHz channel, IEEE 802.11ax station traffic was cut off by legacy stations that achieved greater throughput in the case of enabled network coloring. Such behavior may indicate more frequent access to the channel by older stations with the coloring mechanism enabled, resulting from their lack of understanding of the new rules in the network, as they themselves have not implemented this mechanism.

Table 5 summarizes the Scenario 2. It includes the maximum obtained values of throughput in Mbps for mixed ax(50%) + ac(50%), ax(50%) + n(50%), and ax(50%) + a(50%) stations’ coexistence. These results were obtained for saturated conditions with a BSS coloring mechanism and aggregation disabled and enabled (for different levels of aggregation).

### 5.3. Scenario 3—Coexistence of IEEE 802.11ax and Legacy Stations with Variable Share of Devices

Scenario number three also focused on analyzing the presence of older stations in a network with an IEEE 802.11ax access point. This time, the evaluation of network parameters was focused on changing the proportion of legacy clients, which gradually increased to represent 0%, 25%, 50%, 75%, and 100% of all stations. Simulations were conducted for various aggregation configurations, which are presented as an extension of the basic parameter table (see Table 6). Due to the similarity of network operation in the presence of IEEE 802.11ac/n extensions, the scenario included two extreme legacy extensions, namely IEEE 802.11ac and IEEE 802.11a. The same topology as in scenario number 2 was used in the study.

#### 5.3.1. IEEE 802.11ax + IEEE 802.11ac

The first legacy extension that was studied is the direct predecessor of Wi-Fi 6, IEEE 802.11ac. The network used a channel width of 80 MHz and an MCS value equal to 5. Additionally, several aggregation variants were tested, which include disabled aggregation, default and maximum for the given extension.

**Figure 20:**
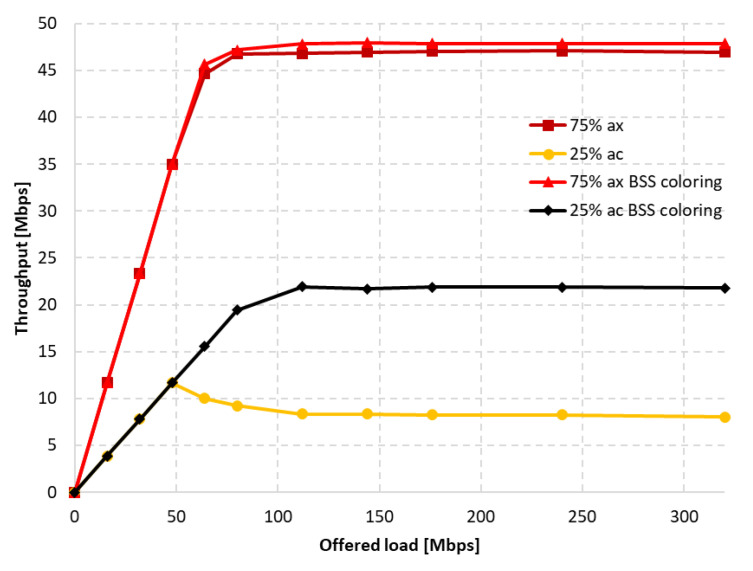
Throughput vs. offered load for mixed ax (75%) and ac (25%) networks without aggregation.

**Figure 21:**
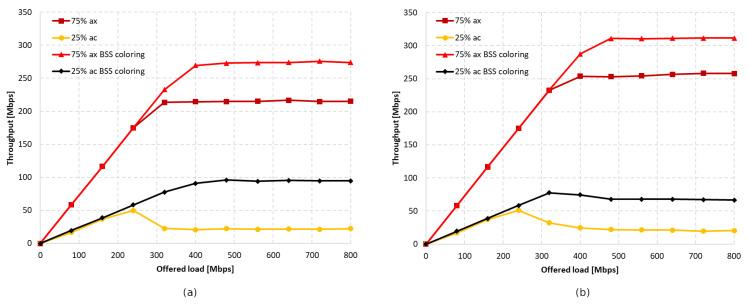
Throughput vs. offered load for mixed ax (75%) and ac (25%) networks: (**a**) default aggregation and (**b**) maximum aggregation.

**Figure 22:**
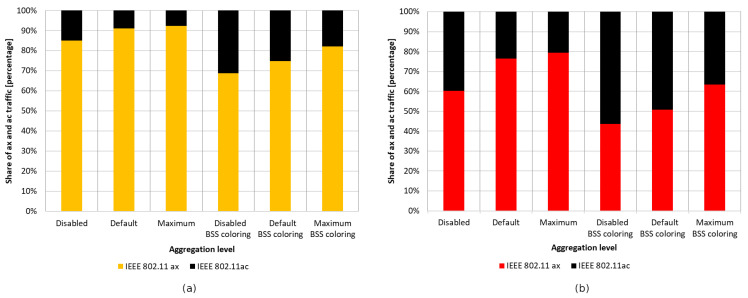
Percentage share of ax and ac traffic for various aggregation levels and configurations: (**a**) 25% of legacy stations and (**b**) 50% of legacy stations.

Figure 20 and Figure 21 show the change in throughput obtained for IEEE 802.11ac legacy stations, depending on the increasing offered traffic with a 25% share of legacy stations. It was observed that, in the absence of aggregation, similar to the previous scenario, the enabled coloring mechanism caused a significant increase in throughput for legacy clients and a small increase for IEEE 802.11ax stations. However, this phenomenon disappears with increasing aggregation, causing an increasing share of transmissions from the IEEE 802.11ax stations in the total network traffic, as shown in Figure 22a. Additionally, the gain resulting from the use of the network coloring mechanism that was cumulatively achieved by both networks was 26%, 55%, and 36%, respectively, for the increasing values of aggregation. The decrease in gain when using the maximum aggregation for a given standard is mainly due to the decrease in throughput for IEEE 802.11ac clients when using the coloring mechanism and network saturation, as presented in Figure 20, which was not observed at lower aggregation values.

The same experiments were conducted for a network with 50% IEEE 802.11ax stations and 50% IEEE 802.11ac stations, but the network behavior has been described in detail in the second scenario. Figure 22b shows the previously unconsidered percentage of traffic distribution for each standard. Once again, it is noticeable that, with the use of a larger frame aggregation, the percentage share of IEEE 802.11ax stations increases.

**Figure 23:**
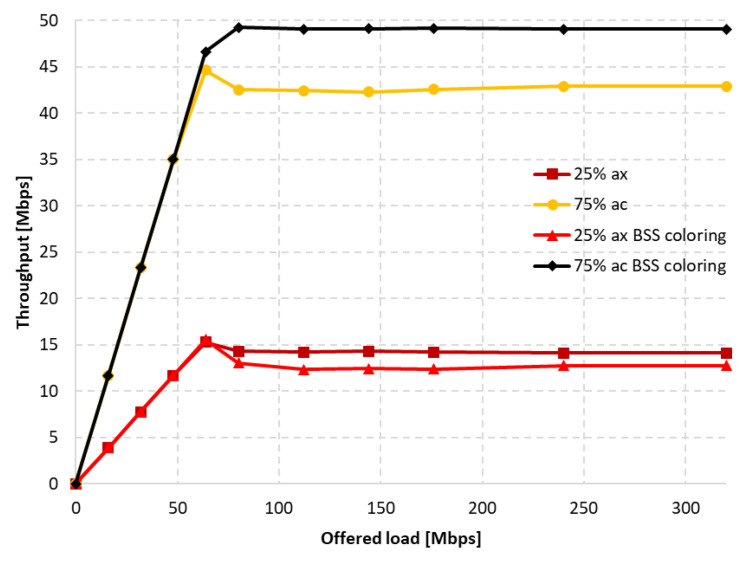
Throughput vs. offered load for mixed ax (25%) and ac (75%) networks without aggregation.

**Figure 24:**
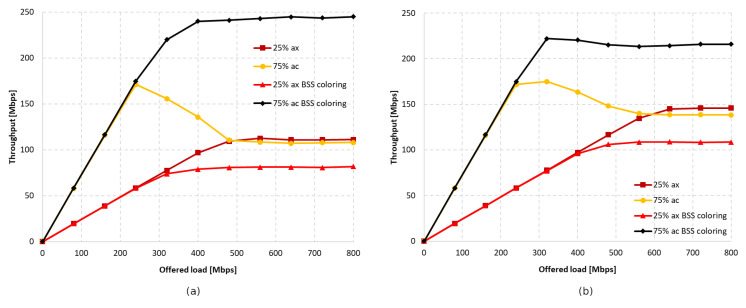
Throughput vs. offered load for mixed ax (25%) and ac (75%) networks: (**a**) default aggregation and (**b**) maximum aggregation.

Next, the simulations were repeated for a ratio of 25% IEEE 802.11ax stations to 75% IEEE 802.11ac stations. Figure 23 and Figure 24 illustrate the throughput as the network becomes increasingly saturated with increasing aggregation sizes.

**Figure 25:**
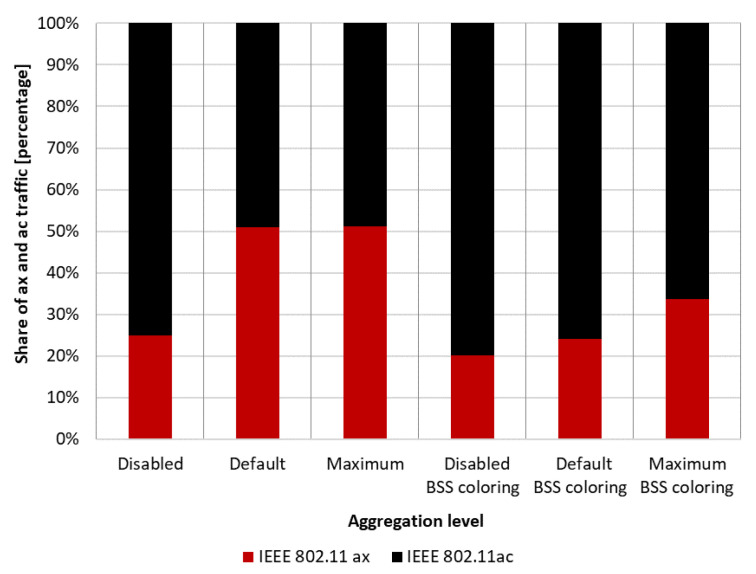
Percentage share of ax and ac traffic for various aggregation levels and configurations and 75% of legacy stations.

It was observed that turning off aggregation leads to a significant domination of legacy traffic, which gradually decreases with increasing aggregated frame sizes. However, unlike the previous experiments conducted using this scenario, network coloring caused a decrease in throughput for IEEE 802.11ax stations in all tested cases. The new mechanism thus led to the increased domination of legacy stationsthe when coloring mechanism was applied, as well as a degradation of IEEE 802.11ax traffic. Once again, however, a trend is observed where the share of IEEE 802.11ax transmissions in the overall network traffic increase with higher aggregation sizes (see Figure 25). In Figure 21, we can also see that the traffic increases linearly as the network saturates for both IEEE 802.11ax and IEEE 802.11ac devices, which stabilizes once the network saturation is reached for IEEE 802.11ax devices and for the 25% ac case with BSS coloring. In the case where BSS coloring is disabled, the traffic decreases to a certain value and then stabilizes. This situation is also somewhat noticeable in Figure 20 for the 25% ac case and in Figure 23 and Figure 24. In Figure 23, a decrease is observed for all curves, except for 75% ac BSS coloring, and in Figure 24 this mainly affects the 75% ac case. This decrease is related to the increased contention in the wireless channel under saturation conditions and the lack of available transmission resources. This results in a larger number of collisions. The scale of the decrease is highly connected with the type of aggregation used. The larger the aggregation size, the longer the IEEE 802.11ax devices block access to the medium for IEEE 802.11ac legacy devices (which is even more evident in the case of IEEE 802.11a extension, as presented in Section 5.3.2), which leads to a decrease in throughput for legacy devices. However, it can be seen that the Distributed Coordination Function (DCF) function operates in a relative stable manner and the decrease occurs only up to a certain value of the realized traffic (this is mainly at the expense of the throughput increase for IEEE 802.11ax standard stations or legacy devices with the BSS coloring mechanism enabled), which is related to the stabilization of the contention window size [30].

#### 5.3.2. IEEE 802.11ax + IEEE 802.11a

The simulation series was also repeated with the coexistence of the oldest extension station operating in the 5 GHz frequency band, which is IEEE 802.11a. MCS 3 was used in the network configuration for the IEEE 802.11ax, because it is closest to the maximum rate for the legacy standard of 54 Mbps. Additionally, a 20 MHz channel was used, which is the only one supported by the tested legacy extension. The network topology remained unchanged and relies on having two access points and four associated stations each.

**Figure 26:**
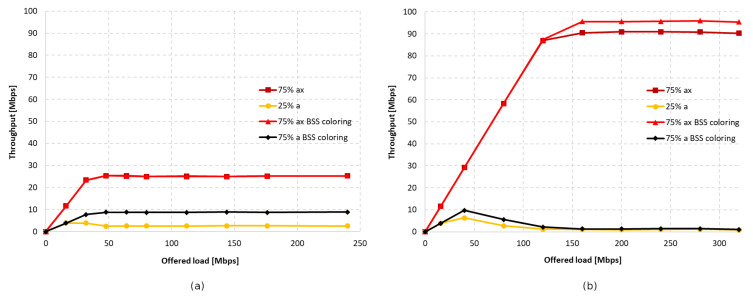
Throughput vs. offered load for mixed ax (75%) and a (25%) networks: (**a**) aggregation disabled and (**b**) maximum aggregation.

Figure 26 shows the change in throughput for no aggregation and maximum aggregation, respectively. As the IEEE 802.11a extension does not have the ability to aggregate frames, this was only configured for the IEEE 802.11ax. In the case of disabled aggregation, a gain of approximately 25% was observed. However, the simulations showed that using frame grouping, and even the presence of a single legacy station, which in this case represents 25% of the clients in the network, leads to much smaller gains from the use of the coloring mechanism, amounting to only 6%.

**Figure 27:**
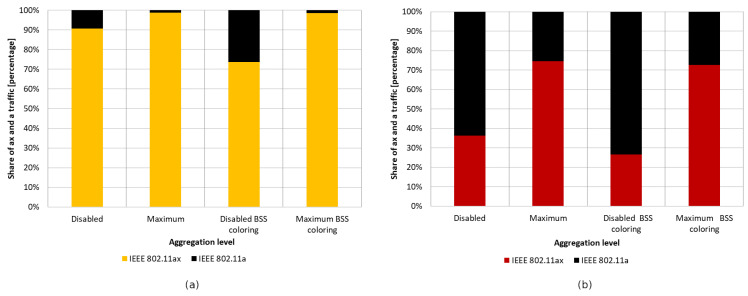
Percentage share of ax and a traffic for various aggregation levels and configurations: (**a**) 25% of legacy stations and (**b**) 50% of legacy stations.

Additionally, as shown in Figure 27a, enabling aggregation resulted in an increased percentage share of IEEE 802.11ax traffic at the cost of a decrease in network gain.

Increasing the proportion of legacy stations (50%–50%) resulted in a significant domination of their traffic, which was again fixed by increasing the aggregation for IEEE 802.11ax stations, as shown in Figure 27b. However, in this case, the total throughput gain in the network is almost zero and amounted to only 2%.

**Figure 28:**
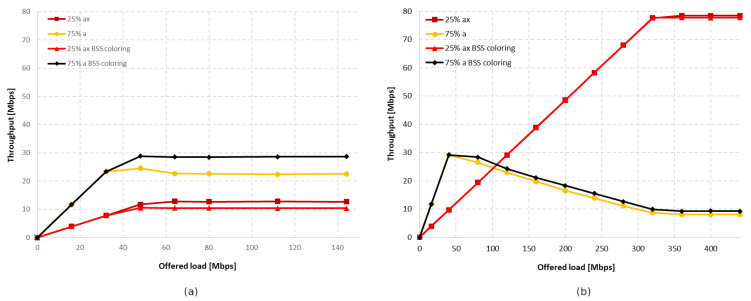
Throughput vs. offered load for mixed ax (25%) and a (75%) networks: (**a**) aggregation disabled and (**b**) maximum aggregation.

Next, the number of legacy stations was increased to 75% of all stations. As shown in Figure 28a, in the case of no aggregation, the network traffic was distributed similarly to the presence of IEEE 802.11ac stations, as described in the previous subsection. The gain from using the coloring mechanism in the entire network was about 10%, and was achieved by legacy clients, while the IEEE 802.11ax stations obtained a reduced throughput. Changes in the network’s behavior are noticeable in Figure 28b, which presents the throughput achieved in the network configuration with the maximum aggregation of IEEE 802.11ax clients. From the simulations, it follows that, despite the advantage of legacy devices, as the network becomes saturated, their throughput rapidly decreases and is displaced by IEEE 802.11ax stations. Their achieved throughput is a kind of extension of the graph in Figure 28a, which represents the throughput in the case of no aggregation. However, although all clients achieved higher throughput, no gain was observed from using the coloring mechanism.

**Figure 29:**
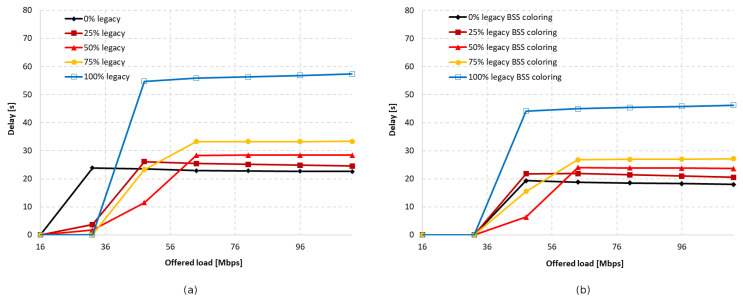
Mean packet delay vs. offered load for mixed ax and a networks: (**a**) BSS coloring disabled and (**b**) BSS coloring enabled.

Additionally, the total delay in the network was analyzed for an increasing percentage of legacy stations when using the coloring mechanism and when it was not used. This was presented as a function of the offered traffic, as shown in Figure 29. The total delay in the network was shown to increase with the presence of a greater number of IEEE 802.11a devices, and 100% of legacy stations caused a 2.5 times higher delay than when they were completely absent. The use of network coloring also contributed to the achievement of smaller network delays in this case.

#### 5.3.3. Summary

**Figure 30:**
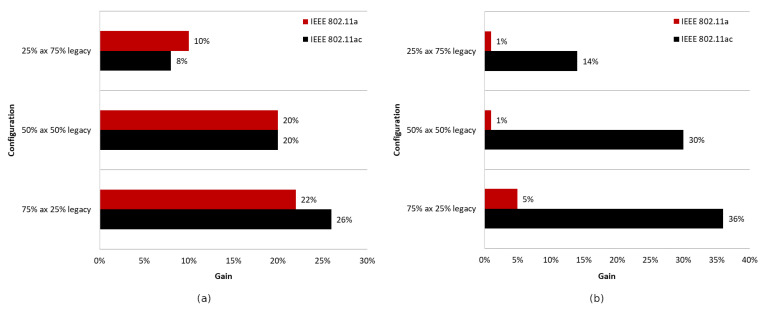
The average percentage increase in throughput for mixed ax and legacy networks: (**a**) aggregation disabled and (**b**) maximum aggregation.

Figure 30 shows the percentage gain achieved through the application of network coloring in situations where older stations, such as IEEE 802.11ac and IEEE 802.11a, coexisted in different network configurations. It was observed that in the absence of aggregation, the percentage gain in achieved throughput is very similar for both legacy extensions. Additionally, as the percentage of older devices in the network increases, there is a gradual decline in the gain, which, as shown in previous sections, is mainly noticeable for IEEE 802.11ac/a devices. This indicates a negative impact of the presence of legacy stations on the network performance, which disrupts the operation of the network coloring mechanism, especially with a smaller share of IEEE 802.11ax stations.

Increasing the number of aggregated frames had a positive effect in the presence of IEEE 802.11ac devices in the network, as this led to an increasing percentage of transmissions by IEEE 802.11ax stations. Additionally, an increased percentage gain in network throughput was observed in this case; however, as in the absence of aggregation, this gradually decreased with a larger number of legacy stations. However, the use of aggregation did not improve network performance when IEEE 802.11ax stations coexisted with IEEE 802.11a devices. Their presence resulted in almost no gain from the application of the coloring mechanism, which decreased to 2% with an increasing number of legacy stations.

The research shows that a network with only IEEE 802.11ax devices always achieved the highest throughput, and the presence of older standard devices led to a reduced throughput. Additionally, the highest gain was observed in the configuration with default aggregation, which is the same for each station. However, the use of maximum aggregation for a given standard led to the highest throughput and an increased percentage share of IEEE 802.11ax traffic, as described in previous subsections.

Table 7 summarizes Scenario 3. It comprises the maximum obtained values of throughput in Mbps for mixed ax(75%) + ac(25%), ax(25%) + ac(75%), ax(75%) + a(25%), and ax(25%) + a(75%) stations coexistence. These values were achieved for saturated conditions with a BSS coloring mechanism and aggregation disabled and enabled (for different levels of aggregation).

## 6. Discussion

It has been demonstrated that the network coloring mechanism strongly depends on the conditions prevailing in the radio channel and the location of individual wireless network devices. The research that was carried out showed that the use of a high OBSS_PD threshold, in this case −64 dBm, is only beneficial in cases with very good radio channel quality and a small number of collisions. When many networks are transmitting nearby on the same channel, using such a high threshold resulted in a decrease in throughput of up to 37%. A dependence was also shown, according to which the optimal OBSS_PD threshold value decreases with an increasing number of networks operating on the same channel. In a network consisting of two access points, the optimal value was −64 dBm, while for three it was −72 dBm, and for configurations with four and seven access points, the most beneficial threshold value was −78 dBm. Additionally, when the number of access points to which separate colors were assigned increased, a higher percentage gain from the use of the coloring mechanism was observed, resulting from an increase in interference, collisions, and the hidden stations that this new mechanism aims to eliminate. The highest gain, amounting to 43%, was achieved for seven networks operating on the same channel. In addition, a negative impact of aggregation was observed when the number of BSS networks transmitting on the same channel was greater than two, and an increase in the number of such networks resulted in an increase in the interference level. The use of aggregation when only two networks were transmitting on the same channel resulted in a throughput gain of up to 79%, while for networks consisting of three, four, and seven access points, the gain in the use of the coloring mechanism was 14%, 7%, and 8%, respectively.

In addition, simulations showed that the presence of older devices in the network disrupts the functioning of the coloring mechanism, resulting in a greater throughput gain for legacy devices. This behavior is likely due to non-compliance with the coloring mechanism rules by legacy devices that do not share this implementation. It was concluded that older extension standard devices have more frequent access to the radio channel, which, in the case of an 80 MHz channel width and no configured aggregation, led to the cutting off of IEEE 802.11ax traffic by legacy clients who, with the coloring mechanism enabled, achieved an 8 Mbps higher throughput. In order to balance the traffic so that they are closer to the throughput ratio without using the coloring mechanism, the use of A-MPDU and A-MSDU aggregation was proposed, resulting in greater gains for IEEE 802.11ax stations. The research also revealed that using the coloring mechanism significantly reduces delay and packet loss, which is particularly important in dense environments and configurations with aggregation, which, in the case of frequent collisions, results in the deterioration of both metrics.

The conducted studies demonstrated that increasing the share of older stations in the network limits throughput. For legacy station to IEEE 802.11ax client proportions of 0%, 25%, 50%, 75%, and 100%, the total throughput with maximum aggregation and coloring mechanisms was 404 Mbps, 378 Mbps, 371 Mbps, 324 Mbps, and 237 Mbps, respectively. Additionally, it was found that even the presence of a small number of the oldest stations operating in the 5 GHz frequency band, such as IEEE 802.11a, with the aggregation of IEEE 802.11ax stations enabled, leads to a complete disruption of the network coloring mechanism, which does not result in any throughput gain. Therefore, when implementing the latest IEEE 802.11ax network, it is recommended to eliminate legacy devices, especially the oldest ones, which can significantly limit the potential of new mechanisms.

The phenomena observed for other MCS values will be very similar; however, the achieved throughput values and the transmission range will change. The use of aggregation mechanisms in the case of IEEE 802.11ax networks using different MCS values for mixed network environments will still improve their performance. In summary, the presence of old stations using the IEEE 802.11a extension in a mixed network will cause the coloring mechanism to operate improperly, resulting in no increase in throughput regardless of the MCS mode used for the IEEE 802.11ax network.

Regrettably, although the conducted analysis shows how to optimize the mixed IEEE 802.11ax networks’ performance, it does not present an automatic method to do so. Research studies have shown that network efficiency is influenced by numerous factors, such as the enabled BSS coloring mechanism, OBSS_PD threshold parameter value, the type and proportion of legacy stations, the size and method of frame aggregation, and the number of access points, as well as their mutual location, and distance from each other. The paper also assumes that the transmission power of stations and access points is constant. However, some research studies [31,32] indicate that to maximize the area network throughput, the level of transmitting power or the carrier sensing threshold, especially in dense networks, should be controlled by an appropriate algorithm. In the real network, rate adaptation algorithms are also used, rather than constant MCS values, which can also affect the optimization process [10,33].

## 7. Conclusions

The improvements proposed by the IEEE 802.11ax standard will play a crucial role in improving transmission efficiency and quality. They offer many changes to the PHY, such as 1024-QAM modulation, 8 × 8 MIMO, and OFDMA technique, which guarantee a significantly higher throughput. However, some mechanisms have been introduced that require appropriate conditions and correct configuration parameters to function properly and provide benefits. One such mechanism is BSS network coloring, which, due to its recent development, has not been thoroughly studied to date. There are some papers devoted to the analysis of the OBSS_PD threshold selection, but the issue of coexistence with older standard extensions has been overlooked.

In this work, an analysis of the IEEE 802.11ax networks’ coexistence with all types of legacy devices, namely IEEE 802.11ac/n/a, is presented. The impact of the BSS coloring mechanism on network performance was evaluated. Three different scenarios with varied numbers and types of legacy nodes were considered. A number of MAC and PHY layer parameters, such as OBSS_PD threshold, channel width, aggregation type and size, were analyzed to optimize the network performance. We found that the performance of the IEEE 802.11ax standard is heavily influenced by the relative location of devices in the network, the presence of legacy nodes, the type of extension that was utilized, and the configuration of multiple mechanisms. Any misconfiguration of these mechanisms can result in a decrease in throughput, as shown in the paper. The research also shows the mechanisms that should be activated and parameter values that should be adopted to increase the efficiency of IEEE 802.11ax networks in which legacy stations of different extensions operate. This allows for the optimization of mixed IEEE 802.11ax networks in a 5 GHz band.

As future work, we would like to analyze network scenarios in closed environments, including ultra-dense networks in 3D buildings, as well as determine the impact of the dual NAV mechanism defined in the IEEE 802.11ax standard, on the network performance. We would also like to propose an optimization algorithm which, depending on the number and types of legacy devices, could select the optimal values of the OBSS_PD threshold and frame aggregation parameters. It would also be interesting to determine whether the QoS metrics of the EDCA function will not deteriorate in the case of IEEE 802.11ax’s coexistence with legacy devices.

## Figures and Tables

**Table 1:** Simulation parameters.

Parameter	Value
Frequency band	5 [GHz]
Station’s transmission power	15 [dBm]
AP’s transmission power	20 [dBm]
Receiver sensitivity	−92 [dBm]
Number of antennas	1
MCS	5 (64−QAM 2/3)
CCA ED threshold	−62 [dBm]
CCA SD threshold	−82 [dBm]
Guard interval	3200 [ns]
RTS/CTS	Disabled
Fragmentation	Disabled
Traffic class	BE
Transport protocol	UDP
Traffic type	CBR
Frame size	1472 [B]

**Table 2:** Scenario specific simulation parameters.

Parameter	Value
OBSS_PD threshold	−64 [dBm]/−72 [dBm]/−78 [dBm]
A-MPDU	0 [B]/65,535 [B]
A-MSDU	0 [B]
Channel width	20 [MHz]
Offered load	500 [Mbps] (saturation)
Number of BSS	2/3/4/7
D1—distance between AP	20–380 [m]
D2—distance between station and AP	2 [m]

**Table 3:** The mean values of throughput in Mbps for Scenario 1.

Number of BSSs	Aggregation	BSS Coloring			
		Disabled	OBSS_PD Threshold		
			−78 dBm	−72 dBm	−64 dBm
2	Disabled	58.00	63.70	67.96	65.49
	Enabled	251.90	252.99	310.53	347.09
3	Disabled	65.94	71.26	78.71	72.14
	Enabled	479.23	488.15	525.04	532.49
4	Disabled	85.65	95.90	100.90	89.54
	Enabled	617.11	632.57	649.24	650.74
7	Disabled	137.79	163.75	169.27	137.92
	Enabled	557.50	560.61	559.44	569.66

**Table 4:** Scenario-specific simulation parameters.

Parameter	Value
OBSS_PD threshold	−72 [dBm]
A-MPDU	0 [B]/65,535 [B] (default)/6,500,631 [B] (max)
A-MSDU	0 [B]/7935 [B] (max)
Channel width	80 [MHz]/40 [MHz]/20 [MHz]
Offered load	0–800 [Mbps] (saturation)
Number of BSS	2
Number of stations in each BSS	4 (2 IEEE 802.11ax + 2 legacy)
D1—distance between AP	240 [m]
D2—distance between station and AP	2 [m]
Legacy standards	IEEE 802.11ac/IEEE 802.11n/IEEE 802.11a

**Table 5:** The mean values of throughput in Mbps for Scenario 2.

Coexistence	IEEE 802.11	Aggregation					
		Disabled		Default		Maximum	
		BSS Coloring Disabled	BSS Coloring Enabled	BSS Coloring Disabled	BSS Coloring Enabled	BSS Coloring Disabled	BSS Coloring Enabled
ax + ac	ax (50%)	32.83	28.39	176.52	175.90	227.62	234.18
	ac (50%)	21.59	36.82	53.76	174.56	57.58	136.56
ax + n	ax (50%)	29.90	22.84	-	-	47.09	46.67
	n (50%)	10.15	20.37	-	-	17.94	40.66
ax + a	ax (50%)	22.38	18.77	-	-	45.09	45.83
	a (50%)	8.05	17.62	-	-	3.22	3.82

**Table 6:** Scenario-specific simulation parameters.

Parameter	Value
OBSS_PD threshold	−72 [dBm]
A-MPDU	0 B/65,535 [B] (default)/6,500,631 [B] (max)
A-MSDU	0 [B]/7935 [B] (max)
Channel width	80 [MHz]/20 [MHz]
MCS	5 (802.11ax)/3 (802.11ax)/54 [Mbps] (802.11a)
Offered load	0–800 [Mbps] (saturation)
Number of BSS	2
Number of stations in each BSS	4
D1—distance between AP	240 [m]
D2—distance between station and AP	2 [m]
Legacy standards	AC/A

**Table 7:** The mean values of throughput in Mbps for Scenario 3.

Coexistence	IEEE 802.11	Aggregation					
		Disabled		Default		Maximum	
		BSS Coloring Disabled	BSS Coloring Enabled	BSS Coloring Disabled	BSS Coloring Enabled	BSS Coloring Disabled	BSS Coloring Enabled
ax + ac	ax (75%)	46.96	47.87	214.89	273.71	257.76	311.47
	ac (25%)	8.04	21.78	22.20	94.75	20.54	66.58
ax + ac	ax (25%)	14.13	12.76	111.07	81.68	145.80	108.67
	ac (75%)	42.88	49.06	107.80	244.97	138.34	215.86
ax + a	ax (75%)	25.24	25.21	-	-	90.27	95.43
	a (25%)	2.58	8.87	-	-	0.79	1.03
ax + a	ax (25%)	12.63	10.39	-	-	78.44	77.75
	a (75%)	22.50	28.69	-	-	8.07	9.27

## Data Availability

The data presented in this study are available on request from the corresponding author.

## Abbreviations

The following abbreviations are used in this manuscript:

AP	Access Point
A-MPDU	Aggregate MAC Protocol Data Unit
A-MSDU	Aggregate MAC Service Data Unit
BE	Best Effort
BSS	Basic Service Set
CBR	Constant Bit Rate
CCA	Clear Channel Assessment
CCA ED	Clear Channel Assessment Energy Detection
CCA SD	Clear Channel Assessment Signal Detection
CCK	Complementary Code Keying
CTS	Clear to Send
DCF	Distributed Coordination Function
DL	Downlink
DSC	Dynamic Sensitivity Control
DSSS	Direct Sequence Spread Spectrum
FHSS	Frequency Hopping Spread Spectrum
GI	Guard Interval
HE ER SU	High-Efficiency Extended-Range Single User
HE MU	High-Efficiency Multi-User
HE SU	High-Efficiency Single User
HE TB	High-Efficiency Trigger-Based
IR	Infra-Red
MAC	Medium Access Control
MCS	Modulation Coding Scheme
MIMO	Multiple-Input Multiple-Output
Multi-TID AMPDU	Multi-Traffic Identifier Aggregated MAC Protocol Data Unit
MU-MIMO	Multi-User MIMO]
NS	Network Simulator
OBSS	Overlapping Basic Service Set
OBSS_PD	Overlapping BSS Preamble Detection
OFDM	Orthogonal Frequency Division Multiplexing
OFDMA	Orthogonal Frequency Division Multiple Access
PAID	Partial AID
PHY	Physical
RTS	Request to Send
RU	Resource Unit
TxBF	Transmit Beamforming
SINR	Signal to Interference and Noise Ratio
UL	Uplink
Wi-Fi	Wireless Fidelity
WLANs	Wireless Local Area Networks
